# Contrasting responses to a climate regime change by sympatric, ice-dependent predators

**DOI:** 10.1186/s12862-016-0630-3

**Published:** 2016-03-15

**Authors:** Jane L. Younger, John van den Hoff, Barbara Wienecke, Mark Hindell, Karen J. Miller

**Affiliations:** 1 Institute for Marine and Antarctic Studies, University of Tasmania, Private Bag 129, Hobart, 7001 Tasmania Australia; 2 Australian Antarctic Division, 203 Channel Highway, Kingston, 7050 Tasmania Australia; 3 Australian Institute of Marine Science, The UWA Oceans Institute, 35 Stirling Highway, Crawley, WA 6009 Australia; 4 School of Biological Sciences, Private Bag 5, University of Tasmania, Hobart, 7001 Tasmania Australia

**Keywords:** Climate change ecology, Bayesian skyline plot, Demographic history, Ecological niche, Resilience, Holocene, *Aptenodytes forsteri*, *Leptonychotes weddellii*

## Abstract

**Background:**

Models that predict changes in the abundance and distribution of fauna under future climate change scenarios often assume that ecological niche and habitat availability are the major determinants of species’ responses to climate change. However, individual species may have very different capacities to adapt to environmental change, as determined by intrinsic factors such as their dispersal ability, genetic diversity, generation time and rate of evolution. These intrinsic factors are usually excluded from forecasts of species’ abundance and distribution changes. We aimed to determine the importance of these factors by comparing the impact of the most recent climate regime change, the late Pleistocene glacial-interglacial transition, on two sympatric, ice-dependent meso-predators, the emperor penguin (*Aptenodytes forsteri*) and Weddell seal (*Leptonychotes weddellii*).

**Methods:**

We reconstructed the population trend of emperor penguins and Weddell seals in East Antarctica over the past 75,000 years using mitochondrial DNA sequences and an extended Bayesian skyline plot method. We also assessed patterns of contemporary population structure and genetic diversity.

**Results:**

Despite their overlapping distributions and shared dependence on sea ice, our genetic data revealed very different responses to climate warming between these species. The emperor penguin population grew rapidly following the glacial-interglacial transition, but the size of the Weddell seal population did not change. The expansion of emperor penguin numbers during the warm Holocene may have been facilitated by their higher dispersal ability and gene flow among colonies, and fine-scale differences in preferred foraging locations.

**Conclusions:**

The vastly different climate change responses of two sympatric ice-dependent predators suggests that differing adaptive capacities and/or fine-scale niche differences can play a major role in species’ climate change responses, and that adaptive capacity should be considered alongside niche and distribution in future species forecasts.

**Electronic supplementary material:**

The online version of this article (doi:10.1186/s12862-016-0630-3) contains supplementary material, which is available to authorized users.

## Background

Given the looming threat of a sixth mass extinction [1], understanding species’ resilience to environmental regime shifts is of immediate concern. How a species responds to environmental change depends broadly on three factors: the continued availability of its preferred ecological niche, its capacity to alter its distribution in response to niche availability, and its ability to adapt to a different ecological niche if necessary, known as niche evolution [2–4]. Current models for estimating species’ responses to environmental shifts assume that ecological niche and habitat availability are the major determinants of a species’ response to climate change [5–7], leading to predictions that species with comparable ecological niches and habitat should respond in similar ways to environmental change. However, individual species have different capacities for dispersal and niche evolution linked to factors including philopatry, mobility, gene flow, genetic diversity and evolutionary rate [3, 4]. Indeed, species with slower rates of niche evolution may be more prone to demographic decline [4]. The influence of such intrinsic factors in species’ climate change responses, and the degree to which they should be considered alongside the recognised factors of ecological niche and habitat availability, remain unclear.

Understanding responses to environmental change is particularly important in the case of predators, which play critical roles in regulating ecosystems [8]. Here we examine two key coastal meso-predators, the emperor penguin (*Aptenodytes forsteri*) and the Weddell seal (*Leptonychotes weddellii*), which both have circumpolar distributions [9, 10] and life-cycles that are closely tied to the seasonality of Antarctic sea ice [11, 12]. Both species use coastal fast ice as a platform upon which to raise their offspring, although the penguin does so during winter, whereas the seal does so during spring [10, 13]. The young of both species are fledged/weaned in late December, coinciding with minimum sea ice extent and maximum productivity [11, 12]. They are both warm-blooded, air breathing species that dive to similar depths and occupy similar trophic levels [14]. Direct competition for prey appears to be minimised by temporal and geographic differences in their foraging habitat [14], with latitudinal overlap strongest in near-coastal areas [15]. Both are prey to apex predators [13, 16, 17], and their generation lengths are similar, estimated at 18/19 years for the penguin/seal [18, 19]. Because of these many similarities, forecasting models based on ecological niche and habitat availability would likely predict that emperor penguins and Weddell seals would respond in similar ways to changes in the Antarctic coastal environment.

Decadal-scale monitoring studies have been conducted in several locations both for emperor penguins and Weddell seals and these indicate populations of both species are changing in response to current environmental change. Declines in emperor penguin numbers have been observed at the Pointe Géologie and Haswell Island colonies, and an entire colony has been lost at the Dion Islands [20–23]. These declines were thought to be the result of climatic or oceanographic impacts on breeding success and/or adult survival [21–23]. However, new evidence suggests that the colony at the Dion Islands may have relocated to an area with more favourable sea ice conditions 190 km away [24] and that the declines at Haswell Island and Pointe Géologie may be the result of emigration as a response to locally poor sea ice conditions [22, 24–26]. For Weddell seals, numbers declined at Anvers Island, Antarctic Peninsula, over the period of 1973 – 2002, concurrent with declines in sea ice, and this species has now all but vanished from the region [6]. Weddell seal numbers have also decreased in McMurdo Sound, Ross Sea since the 1950s, however, in this region, fast ice conditions are relatively unchanged and are unlikely to be the cause of the decline [27]. Rather, it has been proposed that the reduced number of seals in McMurdo Sound could be the result of changes in the distribution and abundance of prey resources, which may have either decreased the survival rates of sub-adults, or caused the seals to emigrate to other areas [27]. Interestingly, a monitoring study at the Vestfold Hills, East Antarctica, showed no downward trend in Weddell seal numbers over the period 1973–2000 [28], despite a local shortening of the sea ice season [29]. To date, the observed population trends of Weddell seals and emperor penguins are regionally variable and not clearly linked to any one environmental driver. Nevertheless, projected declines in their sea ice breeding habitat [30] are expected to be problematic for both species in the future [6, 7, 31].

In the late Pleistocene, following the last glacial maximum (LGM, 26 – 19.5 kya), environmental conditions in Antarctica changed dramatically, providing an ideal scenario to test the climate change induced responses of emperor penguins and Weddell seals over millennia. LGM air temperatures were *ca.* 13 °C colder than the present day [32], the winter sea ice field was approximately double its present size, extending to about 50°S [33] and, unlike today’s seasonality, the LGM sea ice cover was heavy and perennial [34]. Levels of primary productivity were also reduced [35], possibly having a regulatory effect on the upper trophic levels.

A population’s demographic history is encoded in its genome, allowing for a window into its responses to past climate change [36, 37]. We reconstructed the population trajectories both of emperor penguins and Weddell seals over the past 75,000 years, from the late Pleistocene era through the LGM and into the Holocene, using mitochondrial DNA from colonies in East Antarctica. We chose to focus our study on East Antarctica due to the spatial heterogeneity of climate change trends around Antarctica [38], and the high relevance of regional changes in the context of ecological responses. Using Bayesian coalescent inference [36, 39], we analysed changes in effective female population size (*N*_*ef*_) of both species, and assessed patterns of modern genetic diversity and gene flow. In keeping with the current paradigm, we hypothesized that, as species with overlapping spatial distributions and ecological niche, emperor penguin and Weddell seal population sizes may have shown similar trajectories in response to post-glacial warming following the LGM.

## Methods

**Fig. 1:**
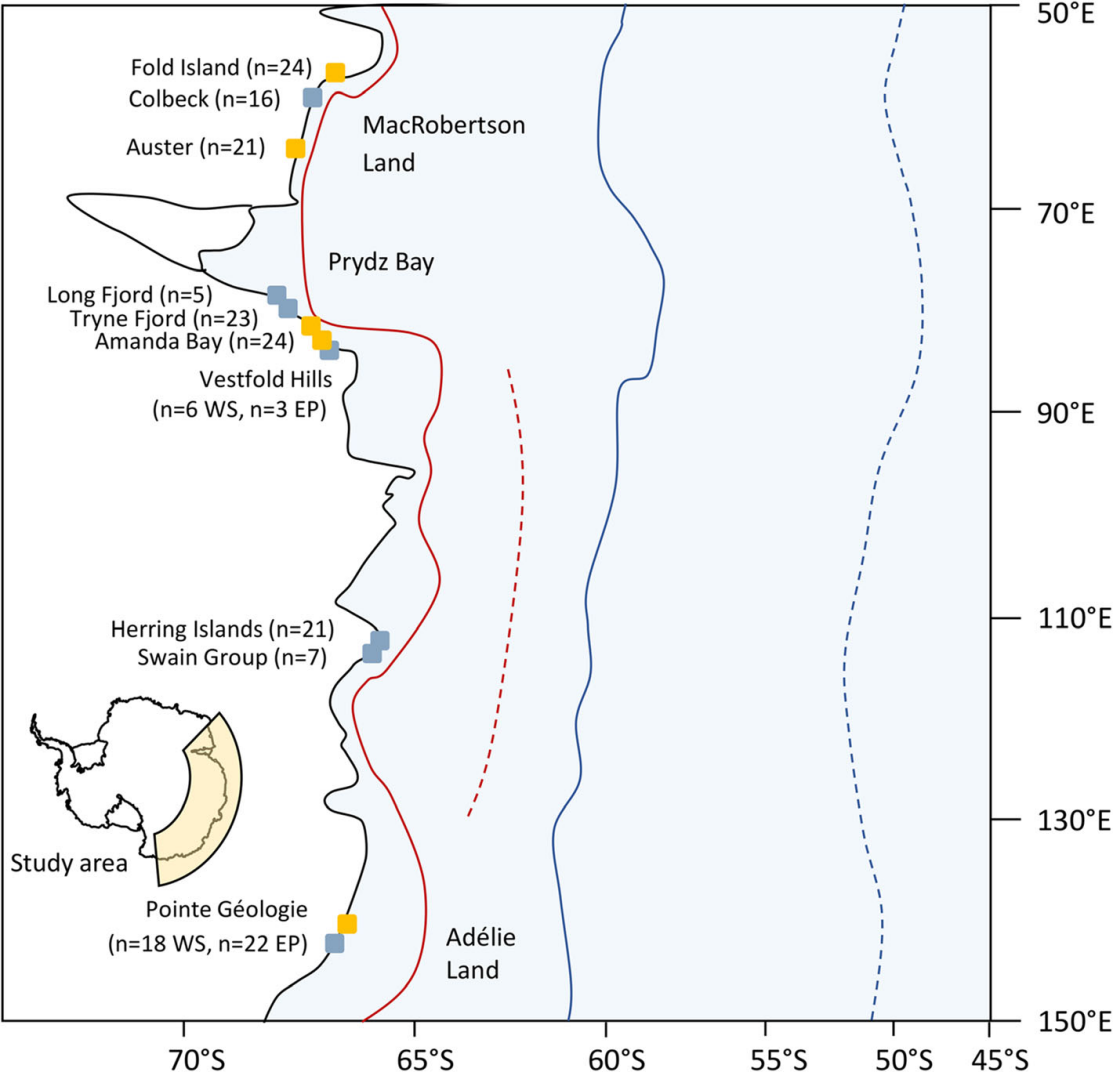
Sampled colony locations and sea ice limits. Yellow/blue boxes indicate emperor penguin/Weddell seal colonies, with the current summer/winter sea ice extents indicated by solid red/blue lines, and the LGM summer/winter sea ice extents by dashed red/blue lines [33]

Genetic material was collected from extant breeding colonies of emperor penguins and Weddell seals in East Antarctica, spanning approximately 4,000 km of coastline (Fig. 1). In this region, Weddell seals and emperor penguins have broadly overlapping breeding distributions. While the emperor penguins require solid fast ice for breeding, the seals prefer breeding sites adjacent to tidal cracks; however, breeding individuals of the two species are often located within a few kilometres of each other across the East Antarctic region. All field activities were conducted under permits issued by the Australian Antarctic Division following independent ethical review. Weddell seal flipper biopsies were collected from 90 individuals across six breeding sites (Table 1; Fig. 1) between 1996 and 2010 and stored at −20 °C. Pectoral muscle biopsies were collected from 91 dead emperor penguin chicks at four colonies (Table 1; Fig. 1) between 1993 and 2013 and stored at −20 °C [40]. DNA sequences from radiocarbon dated sub-fossil remains were included in the demographic history analyses as additional calibration points for the molecular clock. Bones from the sub-fossil remains of three penguins and six Weddell seals were collected in the Vestfold Hills and the radiocarbon ages, expressed here as years BP (i.e. before 1950), of the sub-fossil remains were determined using accelerated mass spectrometry by GNS Science Rafter Radiocarbon National Isotope Centre, New Zealand. The apparent ages were corrected for the marine-carbon reservoir effect [41] using the calibration program Calib7.0 [42]. The corrected ages of sub-fossil remains ranged from 643 to 881 years BP for emperor penguins, and 690 to 1172 years BP for Weddell seals.

**Table 1:** Number of individuals sequenced by species and colony location

	Emperor penguins	Weddell seals
Auster	21	-
Amanda Bay	24	-
Fold Island	24	-
Pointe Géologie	22	18
Tryne Fjord	-	23
Long Fjord	-	5
Herring Islands	-	21
Swain Group	-	7
Colbeck	-	16
*Total extant*	91	90
Vestfold Hills	3	6
*Total sub-fossil*	3	6

DNA was extracted from modern samples with the QIAGEN DNeasy Blood and Tissue Kit following the manufacturer’s protocols. For subfossil samples ~50 mg of bone, taken from the interior of the bone specimens following removal of the outer layers with a scalpel, was decalcified in 0.5 M EDTA/0.001 % Triton X100 at 56 °C for 48 h and then DNA extracted using a standard phenol chloroform protocol with ethanol precipitation. The sub-fossil samples were extracted in a physically isolated laboratory that had never been used before for avian or pinniped samples, with extractions performed inside a laminar flow cabinet to further minimise contamination risk. Negative extraction controls were also used to confirm there was no contamination.

The mitochondrial hypervariable region (HVR) and cytochrome *b* (CytB) were sequenced for all individuals. HVR (491 base pairs (bp); GenBank accessions: KU885090 - KU885185) and CytB (1117 bp; GenBank accessions: KU885226 - KU885321) were amplified and sequenced for modern seals using primers TDKD/L15926 [43, 44] and L-CytB/H-CytB [45] (all primer sequences can be found in Additional file 1: Table S1). HVR (629 bp; GenBank accessions: KP645013-KP645015; KP644913 - KP644958; KP644787-KP644831) and CytB (995 bp; GenBank accessions: KP640871 - KP640873; KP640771 - KP640816; KP640645 - KP640689) were amplified and sequenced for modern penguins using primers F-0225/R-INR [40] and B1/B6 [46]. For the sub-fossil samples we designed novel primers (Additional file 1: Table S1) to amplify short (<150 bp) overlapping fragments in order to improve the success rate of amplification from degraded DNA. The sub-fossil PCRs were done separately from the modern PCRs, and set up in a laminar flow hood in a physically isolated laboratory that is never exposed to PCR products. Negative PCR controls were used for both sub-fossil and extant reactions.

A number of heteroplasmic sites were observed in the emperor penguin HVR sequences, as has been recorded previously in the HVR of the closely related Adélie penguin (*Pygoscelis adeliae*) [47]. These were re-scored manually according to IUPAC ambiguity codes if the secondary peak was >40 % of the height of the primary peak in both forward and reverse sequences.

Arlequin v3.5 [48] was used to calculate summary statistics by colony and genetic region (Additional file 1: Table S2), to quantify genetic differentiation (F_*ST*_) between pairs of colonies and to perform analyses of molecular variance (AMOVA), with 10,000 random permutations of the data to determine the statistical significance of the departure from panmixis.

Demographic reconstructions were done using the extended Bayesian skyline plot (EBSP) method in BEAST v1.8 [39, 49]. For molecular clock calibration of the emperor penguin analyses, the HVR substitution rate prior was specified as a lognormal distribution around a mean value of 0.55 substitutions/site/Myr (SD = 0.15), to reflect the substitution rate of the HVR in the closely related Adélie penguin [47]. In the absence of a published substitution rate for CytB in penguins, we used a uniform prior of 5x10^−4^ to 5x10^−1^ substitutions/site/Myr with a starting value of 2x10^−2^ [50]. The corrected radiocarbon ages of the sub-fossil samples were input as tip dates (i.e. ages were assigned to the sequences) for additional calibration of the molecular clock. Based on these priors, the emperor penguin substitution rates were estimated during the analysis.

There are no reliable published estimates for the Weddell seal substitution rates. We therefore conducted initial BEAST analyses to estimate substitution rates for HVR and CytB using an expanded dataset of 136 Weddell seals. The additional 40 seals, which we included to incorporate as much genetic variation as possible in our estimate, were sourced from other locations in East Antarctica (*n* = 19) and the Ross Sea (*n* = 21) (GenBank accessions: KU885186 - KU885225 and KU885322 - KU885361). The 19 individuals from East Antarctica were excluded from our estimates of population structure and demographic history because the sample sizes were less than five per colony, which we used as a minimum cut-off for this study. HVR and CytB sequences for the Weddell seal’s closest relative, the leopard seal (*Hydrurga leptonyx*) (GenBank HLU03590 and AY377323)*,* were incorporated and the divergence time of the two species (2.89 MYA; 95%CI = 1.84-3.97 MYA) [51] was used as a node calibration. The root height prior was specified as a normal distribution around a mean of 2.89 million years (SD = 0.65). The corrected radiocarbon ages of the sub-fossil samples were input as tip dates for additional calibration of the molecular clock. Based on these initial priors, the Weddell seal HVR and CytB substitution rates were estimated following the BEAST conditions outlined below. For the Weddell seal demographic reconstructions, the substitution rate priors for HVR and CytB were specified as lognormal distributions around a mean value of 0.114 substitutions/site/Myr (SD = 0.15) and 0.0285 substitutions/site/Myr (SD = 0.25) respectively, to reflect our estimates from the initial analyses.

For all BEAST analyses, including the initial estimation of Weddell seal substitution rates, the datasets were partitioned into HVR and CytB, with nucleotide substitution models of HKY [52] with four gamma categories for HVR of both species and for Weddell seal CytB, and TN93 [53] for emperor penguin CytB, to reflect the optimal models selected by jModelTest [54]. We used the EBSP tree prior [39] with a strict molecular clock for all analyses. The posterior distributions of substitution rates and effective population size through time were generated using the Markov chain Monte Carlo sampling procedure, implemented in BEAST, which was run for 80 – 120 million generations, depending on the dataset size, with samples drawn every 6000 steps and the first 10 % discarded as burn-in. Tracer v1.5 was used to visualise the sampling trace and to check the effective sample size values (ESS) to confirm convergence, with all ESS values >200. Three independent BEAST analyses with different random number seeds were performed for each dataset to ensure reproducibility of the posterior distributions.

The population size parameter of the demographic model (*N*_e_*tau) was converted to female effective population size (*N*_*ef*_) by dividing the parameter by generation length (*tau*), where *tau* = A + [S/(1-S)], A = average age of maturation and S = annual survival probability of adults. *Tau* was estimated at 19 years for Weddell seals based on an average female maturation of 7.62 years and an annual survival rate of 0.92 for breeding females [18]. Our estimate of *tau* for emperor penguins was 18 years, based on an age of first breeding of 5.5 years and an annual adult survival rate of 0.925 ([19] and references therein). Note that in our previous publication [40] we used the minimum estimate of emperor penguin generation length, 14 years [19], and have now adjusted this to the mean estimate of 18 years to be directly comparable with the Weddell seal generation length, which is also a mean estimate. It should be noted that any variance in the estimate of generation length, which is difficult to estimate accurately in the case of emperor penguins because demographic data are limited to a single site [21], will only affect the absolute values of *N*_*ef*_ in our results and has no bearing on either the timing or magnitude of the abundance increases reported.

To confirm that heteroplasmies in the emperor penguin HVR were not causing a false signal of expansion we also estimated demographic history using CytB alone for emperor penguins. The analysis settings were as described above, with the exception of the tree prior, which was specified as Bayesian skyline plot instead of EBSP. The signal of expansion from CytB alone (Additional file 1: Table S3) was consistent with the signal from HVR and CytB combined.

**Fig. 2:**
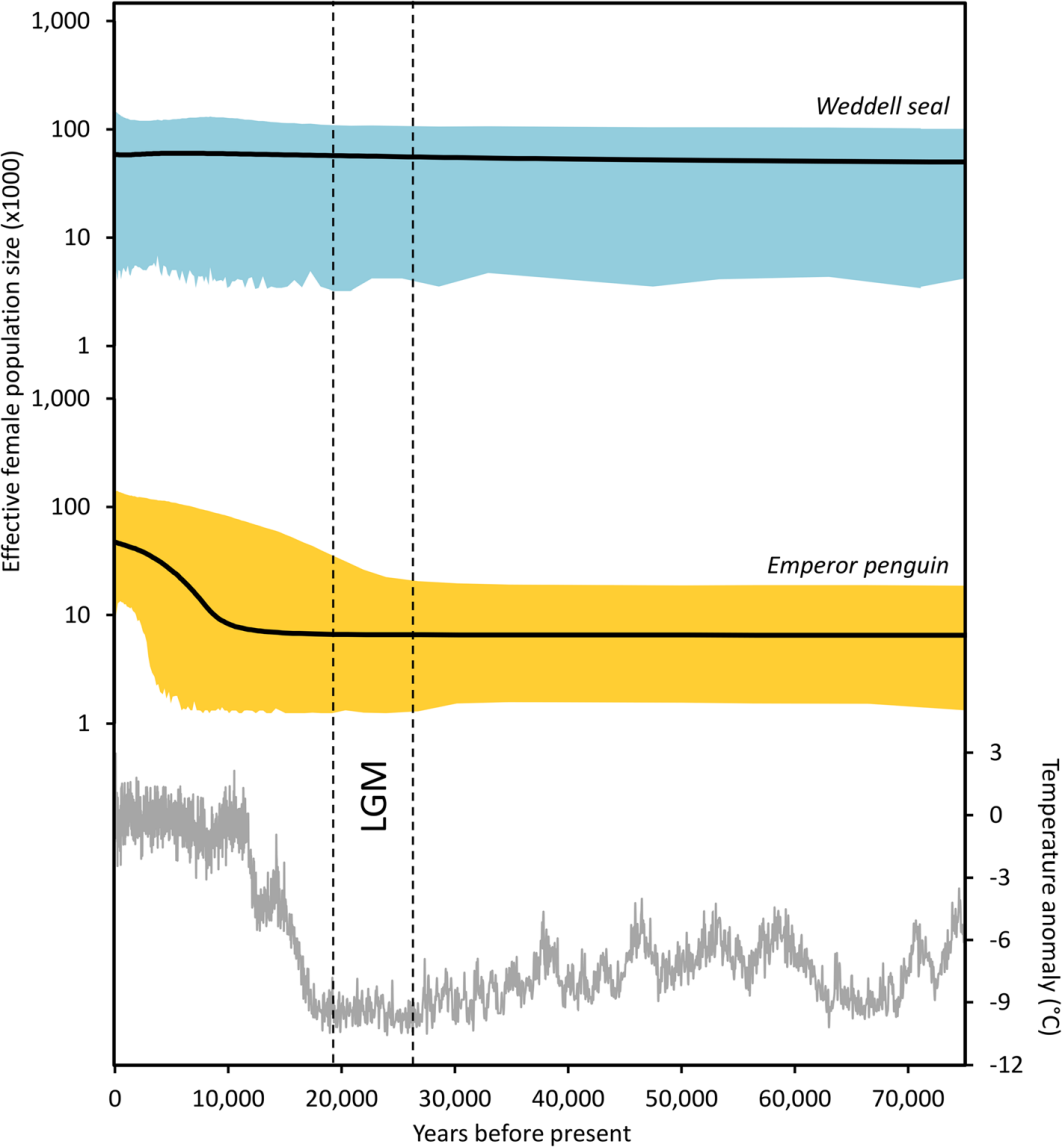
Population trajectories of East Antarctic Weddell seals and emperor penguins over the last 75,000 years. Extended Bayesian skyline plots showing the change in effective female population size (*N*_*ef*_) of Weddell seals (top) and emperor penguins (middle), with the black line indicating the median estimate and colour blocks representing the 95 % highest posterior density interval. The East Antarctic temperature anomaly (the difference from the average of the last 1,000 years) [32], is shown underneath

Population structure, such as that of Weddell seals, can have confounding effects on Bayesian skyline plots, as an assumption of the method is that the population under consideration is panmictic [39, 55]. The main danger is that Bayesian skyline plots of structured populations can show false signals of population decline when, in fact, there has been no population change [55]. The most appropriate sampling strategy for structured populations is the ‘pooled sampling strategy’, wherein 2–20 individuals are sampled from 2–20 demes (populations) [55]. This pooled sampling strategy, which we have applied here for Weddell seals, has been shown to be the most reliable way to capture true signals of population decline and expansion for structured populations [55]. The use of this sampling strategy, combined with the lack of observed population decline (Fig. 2), suggests that the reported population trajectory for Weddell seals is reliable.

## Results

**Fig. 3:**
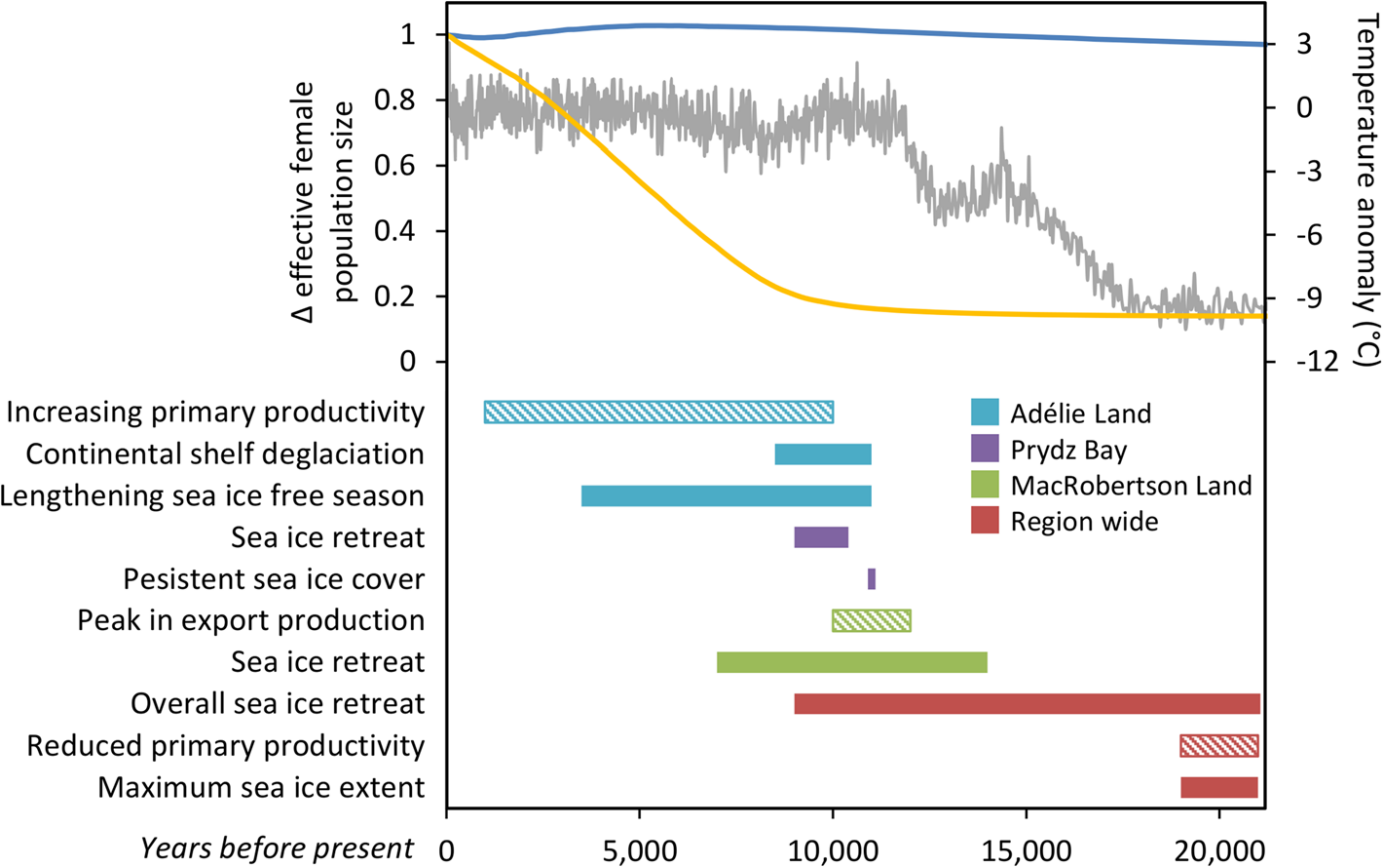
Post-glacial and Holocene environmental changes by sub-region compared to abundance trajectories of Weddell seals and emperor penguins. Median estimate of the change in effective female population size (*N*_*ef*_) relative to today; the yellow/blue lines indicate emperor penguins/Weddell seals. The grey line indicates the East Antarctic temperature anomaly (the difference from the average of the last 1,000 years) [32]. Coloured boxes indicate approximate period of environmental changes including; deglaciation [71, 72], changes in sea ice cover [33, 34, 73] and primary production based on export production indices [35, 57–59]

Our coalescent based 75,000 year reconstruction of *N*_*ef*_ for East Antarctic emperor penguins and Weddell seals showed clear differences in their population trajectories (Fig. 2). Both population trajectories began with a period of relative stability during climate cooling-warming cycling events during the mid to late Pleistocene and the LGM (Fig. 2). Following the LGM, early in the Holocene epoch, an expansion in the population of East Antarctic emperor penguins is indicated by a 5.7 fold increase in *N*_*ef*_ from *ca.* 10,000 years ago to the present (median estimate, Fig. 2). Over precisely the same 75,000 years of periodic climate and environmental fluctuation, Weddell seal *N*_*ef*_ remained relatively unchanged (Figs. 2, 3).

Present day emperor penguin and Weddell seal populations have very different genetic structures; the seals displayed an order of magnitude more genetic differentiation among colonies than the penguins (Tables 2, 3). Emperor penguins breeding along the 4,000 km coastline of East Antarctica constitute a single panmictic population based on mitochondrial DNA (overall F_ST_ = 0; *p* = 0.553; pairwise F_ST_ amongst localities *p* > 0.05; Table 3). This degree of admixture suggests there are currently few, if any, physical barriers to emperor penguin dispersal along the East Antarctic coastline. Conversely, East Antarctic Weddell seal populations are significantly structured (overall F_ST_ = 0.12602; *p* < 0.001) with 10 of the 15 pairwise comparisons showing significant genetic differentiation (Table 2). Small scale spatial structure is strong, with genetically distinct colonies located in embayments separated, in some cases, by as little as 10 km (e.g. Swain Group vs. Herring Islands; pairwise F_ST_ = 0.32629, *p* < 0.001). It should be noted, however, that two of our Weddell seal study colonies, Swain Group and Long Fjord, have low samples sizes (n < 10), therefore the pairwise F_ST_ comparisons for these sites are likely to be less precise than the other locations. The apparent isolation of the Long Fjord, Tryne Fjord and Herring Islands colonies (Table 2) has not reduced Weddell seal genetic diversity relative to colonies at other locations (Additional file 1: Table S2).

**Table 2:** Pairwise genetic differentiation (F_ST_) between Weddell seal colonies

	Tryne Fjord	Long Fjord	Herring Is	Swain Group	Colbeck
Long Fjord	0.1702**				
Herring Is	0.1991***	0.3410**			
Swain Group	0.0832*	0.1248*	0.3263***		
Colbeck	0	0.1448*	0.1878***	0.0534	
Pointe Géologie	0	0.1527**	0.1852***	0.0469	0

*p-*values are denoted as * *p* < 0.05, ** *p* < 0.01, *** *p* < 0.001

**Table 3:** Pairwise genetic differentiation (F_ST_) between Emperor penguin colonies

	Auster	Amanda Bay	Pointe Géologie
Amanda Bay	0		
Pointe Géologie	0.01175	0.01092	
Fold Island	0	0	0

All *p*-values were greater than 0.05, indicating no significant differentiation

The extant genetic diversity was extremely high for the emperor penguin HVR, with 90 haplotypes recorded out of the 91 individuals sequenced; the mean number of pairwise differences between haplotypes was 17.89 ± 8.009. Genetic diversity was much lower based on HVR in Weddell seals (5.102 ± 2.49) and for cytochrome *b* (CytB) of both species (seals = 5.658 ± 2.73; penguins = 2.857 ± 1.51) (detailed summary statistics are provided in Additional file 1: Table S2). The mutation rates, expressed here as substitutions/site/Myr with standard error, were higher in emperor penguins (HVR = 0.760 ± 4.55x10^−3^, CytB = 3.88x10^−2^ ± 3.58x10^−4^) than in Weddell seals (HVR = 0.114 ± 1.76x10^−3^, CytB = 2.85x10^−2^ ± 4.48x10^−4^).

## Discussion

Genetic data have revealed that emperor penguins and Weddell seals had very different population responses to post-glacial climate warming, despite their apparently overlapping habitat requirements, trophic levels and distributions. These results highlight the importance of small differences in ecological niche and the influence of adaptive capacity in shaping species’ responses to environmental change.

### Ecological niche

Warming temperatures at the end of the Pleistocene saw the Earth’s environment shift away from the glacial conditions of the LGM into a period of retreating ice sheets, deglaciation, rising sea levels, increased seasonality of a decreasing sea ice cover and increased primary production (Fig. 3 and references within). These environmental changes occurred asynchronously in East Antarctica (Fig. 3). Given their adaptation to the cold and apparent obligate requirement to use sea ice as a breeding platform, it has been hypothesised that emperor penguins may have prospered during the LGM [56], although our genetic data indicate that past emperor penguin populations were not favoured during periods of glaciation [40]. Indeed, the *N*_*ef*_ for emperor penguins only increased to present day values following the end of the LGM. In contrast, while Weddell seals are also dependent on sea ice cover for breeding, their *N*_*ef*_ did not change following the LGM. Given the genetic differentiation detected among extant Weddell seal populations, it is possible that if a distinct lineage of seals were extirpated during the Pleistocene it may not be detected by our EBSPs. However, our pooled sampling strategy would have detected any population size changes among the ancestors of the extant East Antarctic seals and it is unlikely that the LGM would have differentially affected Weddell seal lineages to such a degree that one was extirpated while another was unaffected. We conclude, therefore, that cold temperatures and an increased sea ice field during the LGM did not positively influence *either* species’ population size, but the *N*_*ef*_ for emperor penguins has responded positively to other habitat changes during the subsequent post-glacial warming period.

Owing to increases in primary production during the post-glacial period [35, 57–59], it is likely that prey resources also increased at this time, which may have led to the observed increase in emperor penguin numbers. However, based on the static Weddell seal *N*_*ef*_ trend, Weddell seals appear to have been unaffected by the same changes in prey availability. This is unexpected as the two predators currently forage at the same trophic level within the meso-pelagic niche [60, 61]. While current prey choice does not necessarily reflect historical foraging behaviour, both species are generalist foragers today and should, in theory, be flexible in the prey species that they consume. While they do consume similar prey, the seals tend to forage over the continental shelf whereas the penguins forage farther north in the pelagic zone [15]. This fine scale difference in preferred foraging habitat may partially explain the differing historical trends, as the pelagic system has more capacity to grow compared to the spatially restricted shelf habitat, a difference that could have favoured emperor penguins. The penguins might also have been more capable of responding to fluctuations in food availability based on their physiology. Juvenile Weddell seals operate at the edge of their physiological diving ability and cannot increase their effort to exploit deeper foraging areas during periods of reduced prey availability [14]. Seals also cannot reduce their energy requirements by abandoning chicks, as adult emperor penguins can [14]. Furthermore, the energy requirements of an individual emperor penguin are substantially less than that of a Weddell seal, owing to their smaller body size. These differences may have allowed emperor penguins to prosper relative to Weddell seals during times of food scarcity.

The depletion of apex predators may result in an increased abundance of meso-predators [8], hence, changes in predation pressure during the Holocene could have affected population trajectories of emperor penguins and Weddell seals. The killer whale (*Orcinus orca*) is an apex predator that preys upon both emperor penguins and Weddell seals [17]. However, killer whale numbers rapidly increased during the Holocene following a population bottleneck during the LGM [62]. It is therefore unlikely that reduced predation by killer whales during the Holocene was responsible for the increase in emperor penguin population size. Conversely, the increase in emperor penguins, a prey resource of killer whales, may have been a contributing factor to the increase in killer whale numbers during the Holocene.

### Adaptive capacity

Dispersal facilitates range shifts and promotes gene flow among breeding sites, which can replenish the gene pool of a population with new, potentially adaptive alleles [3]. We found that emperor penguin colonies across East Antarctica are not genetically differentiated (Table 3), suggesting that there is ongoing gene flow among colony sites within the region. This finding also indicates that there are no substantial barriers to dispersal across East Antarctica for emperor penguins, suggesting that the species may be able to emigrate in line with favoured conditions. Our finding of high mobility in emperor penguins is supported by recent observations of colony relocations [24, 25]. A continent-wide genetic study of emperor penguin population structure also found that the penguins were panmictic across *ca.* 8,000 km of coastline [40]. Together, these studies strongly refute total philopatry among emperor penguins, and this must be taken into account in population forecasting studies for emperor penguins, which typically consider breeding colonies as isolated units [7].

Weddell seals, on the other hand, have genetically distinct breeding colonies with a high degree of philopatry, consistent with previous findings of strong site fidelity [63–65]. Our finding is based on mitochondrial DNA and therefore represents philopatry of females only, however, Cameron et al. (2007) found no difference in the degree of philopatry between male and female Weddell seals, therefore the patterns of genetic structure should be similar for both sexes. The relative isolation of Weddell seal colonies means that they may be more susceptible to genetic drift and loss of adaptive variation, and are less likely to range shift.

We found that the mitochondrial mutation rates for emperor penguins were greater than for Weddell seals. The faster molecular evolutionary rate of emperor penguins compared to Weddell seals is atypical, as mammals general have higher evolutionary rates than birds [66]. This could indicate an increased adaptive capacity of emperor penguins compared to Weddell seals, as higher rates of molecular evolution in mitochondrial DNA are correlated with higher rates of evolution in nuclear genes [67]. Such a notion could be confirmed in the future by comparing evolutionary rates in potentially adaptive loci, which would allow a more accurate estimation of adaptability.

As the East Antarctic environment changed during the Holocene, the high dispersal ability of emperor penguins may have facilitated range expansion and the establishment of new breeding colonies, leading to increases in population sizes and genetic diversity. On the other hand, it must be considered that the stability of Weddell seal *N*_*ef*_ throughout several climate oscillations suggests that Weddell seals are robust to environmental changes, and may have a broader range of climatic tolerance compared to emperor penguins. Whether the environmental conditions in East Antarctica over the past 75,000 years have been optimal for Weddell seals is unknown. An investigation of Weddell seal population sizes further back in time, throughout earlier climate regime shifts, could determine whether different climate scenarios are more or less favourable for Weddell seals. This could be achieved using the pairwise sequentially Markovian coalescent method, which allows for the estimation of population sizes over deeper time scales [68]. Our finding that the Weddell seal population size did not decline during the LGM, the post-glacial period, or the Holocene suggests that the species was able to withstand environmental change, even if they did not rapidly expand during the Holocene as emperor penguins did.

### Adaptive capacity and contemporary climate change

Emperor penguins are sensitive to changes in sea ice conditions over decadal and yearly timescales [7, 21, 31]. In recent years, when sea ice conditions have been sub-optimal, emperor penguins have demonstrated adaptive behaviour in relation to their breeding strategy by relocating colonies from their traditional sea ice breeding habitat onto ice shelves [26]. This behaviour was first observed in 2008, and there are now four known ice shelf breeding colonies [26]. This adaptation is consistent with our findings that emperor penguins have shown high adaptive capacity in the past. Emperor penguins have also shown plasticity in the locations of their colonies, with several cases of relocation of entire colonies coincident with sub-optimal sea ice conditions at the former sites [23–25]. In addition to the movement of entire colonies, there is also evidence that emperor penguin individuals emigrate to more favourable locales, again as a response to sub-optimal sea ice conditions [22, 24]. The findings of colony relocation and emigration show that the dispersal ability of emperor penguins, which our genetic data has shown to be great, is aiding in their response to contemporary unfavourable environmental change, and suggests that this same ability may have facilitated their expansion in the early Holocene.

A 30-year study of Weddell seals has shown that they can adopt a flexible breeding strategy when environmental conditions are sub-optimal, as a means of demographic buffering [69]. This strategy was observed in response to the calving of a large iceberg that disrupted local sea ice conditions and prey availability for several years [70]. The adjacent Weddell seal colony displayed an overall reduction in reproductive rates, however, survival rates were unaffected, and high reproductive rates were re-established directly after the end of the perturbation [70]. Whether demographic buffering enabled Weddell seals to maintain constant population sizes over the past 75,000 years is currently unknown. Relocation of colonies, or emigrations away from sub-optimal breeding locations, have not been documented for Weddell seals under contemporary climate change. This is consistent with our genetic data, which indicates that Weddell seals have low rates of gene flow among colony sites and are not widely dispersive. Interestingly, decadal-scale monitoring studies have demonstrated declines in Weddell seals at both the Antarctic Peninsula [6] and Ross Sea [27], but not in East Antarctica [28], despite local declines in sea ice [29]. The static decadal population trend in East Antarctica is consistent with our millennial trend for Weddell seals. Overall, the responses of East Antarctic Weddell seals and emperor penguins to contemporary climate change are in line with their population trajectories following the glacial-interglacial transition, demonstrating that species’ responses to past climate regime shifts may be good predictors of their future responses to climate warming.

## Conclusions

Our study has revealed the contrasting responses of two iconic Antarctic meso-predators to past climate warming. Differing adaptive capacities and/or fine-scale niche differences likely played a major role in the responses of these predators to environmental change. This is noteworthy, given that broad ecological niche and distribution have been used to forecast declines of several marine predators, without regard to adaptive capacity or fine-scale niche differences. Individual species are likely to respond quite differently to changing habitats and generalisations based on apparent ecological niche may be misleading. Dispersal ability, gene flow and evolutionary rate may be indicators of robustness to climate change, and we suggest that genetic indices of population subdivision, diversity and demographic history are included in future risk analyses for predator species.

### Availability of supporting data

The nucleic acid sequences supporting the results of this article are available in the GenBank repository, accession numbers KP645013 - KP645015; KP644913 - KP644958; KP644787 - KP644831; KP640871 - KP640873; KP640771 - KP640816; KP640645 - KP640689; KU885090 - KU885361.

## Additional file

Additional file 1: Supplementary materials. Table S1. Primer sequences. **Table S2.** Summary statistics of Weddell seal and emperor penguin colonies by genetic region. **Table S3.** Bayesian skyline plot for emperor penguins based on cytochrome b alone. (DOCX 246 kb)
