# Supplementary material for: Contrasting responses to a climate regime change by sympatric, ice-dependent predators
Source: BMC Evol Biol. 2016 Mar 15;16:61. doi: 10.1186/s12862-016-0630-3 (PMC5477764; doi:10.1186/s12862-016-0630-3)
Supplement: Supplementary file 1 — Table S1. Primer sequences. Table S2. Summary statistics of Weddell seal and emperor penguin colonies by genetic region. Table S3. Bayesian skyline plot for emperor penguins based on cytochrome b alone. (DOCX 246 kb) [file 12862_2016_630_MOESM1_ESM.docx]

**S1. Primer sequences**

| Species | Primer name | Primer sequence 5’ – 3’ |
| --- | --- | --- |
| EP | F-0225 | GGAACCTCCCAAAGAGTACCA |
| EP | RINR | CCAACCAGATGTATCGGTGA |
| EP | HVR-R1 | TGAAAGTATTGCTTTACGTATCCTT |
| EP | HVR-F2 | AAGGATACGTAAAGCAATACTTTC |
| EP | HVR-R2 | AGGAGTAATTGTTGAGTACATGACA |
| EP | HVR-F3 | TCATGTACTCAACAATTACTCCTG |
| EP | HVR-R3 | TCACGTGAGAAGACCGACTAA |
| EP | HVR-F4 | ATCTCCTGAGGCGCTAGCTT |
| EP | B1 | CCATCCAACATCTCAGCATGATGAAA |
| EP | B6 | CCATCCAACATCTCAGCATGATGAAA |
| EP | CytB-F1 | ACTGCAGACACAACCCTAGC |
| EP | CytB-R1 | GGGAAGAGGATCAGGAGGGT |
| EP | CytB-R1 | AATGATGCTCCGTTTGCATGTAGGTT |
| EP | CytB-F2 | ACACATGCCGAAACGTACAG |
| EP | CytB-R2 | GTAGCCTACGAAGGCGGTTG |
| EP | CytB-F3 | GAAACCTGAAACACAGGCATT |
| EP | CytB-R3 | CGGGTTAATGTGGGGTTGT |
| EP | CytB-F4 | CTCAGCCATCCCTTACATTG |
| EP | CytB-R4 | TTGTGGAGTAGTAGGGGTGGA |
| EP | CytB-F5 | CAAATAACCCACTGGGCATC |
| EP | CytB-R5 | TCATTCTGGTTTGATGTGTGG |
| EP | CytB-F6 | CCAGCAAACCCACTAGTCAC |
| EP | CytB-R6 | GGGCTCAGAATAGGAGTTGG |
| EP  WS  WS  WS  WS  WS  WS  WS  WS  WS  WS  WS  WS  WS  WS  WS  WS  WS  WS  WS  WS | CytB-F7  TDKD  L15926  L-CytB  H-CytB  Csec2_R  Csec1_R  CytBSec1_F  CytbSec1_R  CytbSec2_F  CytbSec2_R  CytbSec3_F  CytbSec3_R  CytbSec4_F  CytbSec4_R  CytbSec5_F  CytbSec5_R  CytbSec6_F  CytbSec6_R  CytbSec7_F  CytbSec7_R | ATAGCTTTCCGCCCTCTCT  CCTGAAGTAGGAACCAGATG  TCAAAGCTTACACCAGTCTTGTAAACC  AGGCGTCGAAGCTTGACATGAAAAGCCATCGTTG  CGAATTCCATTTTTGGTTTACAAGAC  TCATATATAACATCACTTTCACTGTGC  CAACCACTTTATGTGCATGCTT  TGACATGAAAAATCATCGTTGT  CGGCAGATGTGTGTAACTGA  CAGGCCTATTCCTAGCCATACA  TGTGAATGTGTAGGAGCCGTA  GGAGCATCCATATTCTTCATCTG  CATTGTACTAAGTCAGTTCCGATGT  AAATATCATTCTGAGGAGCAACC  GGGGTGAAATGGGATTTTGT  CACGAGACAGGATCCAACAA  TTGCGTAGGCAAATAGGAAA  CCACATATCAAACCCGAATG  ATGTAGGGGTGTTCGACTGG  TCAGCCAATGCTTATTCTGACT  TGTTCTCCGTTTTTGGTTTACA |

**S2. Summary statistics of Weddell seal and emperor penguin colonies by genetic region**

Overall values for Weddell seals and Emperor penguins refer to extant individuals only, with sub-fossil individuals shown separately. Number of individuals (n), nucleotide diversity averaged over loci (π), number of unique haplotypes (N_H_), mean number of pairwise differences between haplotypes (pairwise) and substitution rate in substitutions/site/Myr (Rate).

|  | | | n | HVR    π | | | N_H_ | | Pairwise | | | Rate | | CytB    π | | N_H_ | Pairwise | Rate | |
| --- | --- | --- | --- | --- | --- | --- | --- | --- | --- | --- | --- | --- | --- | --- | --- | --- | --- | --- | --- |
|  | |  |  |  | |  | |  | | |  | |  |  |  |  |  |  |  |
| Tryne Fjord | | 23 | 0.010 | ± 0.006 | | 11 | | 4.865 ± 2.462 | | |  | | 0.005 | ± 0.003 | 10 | 5.304 ± 2.657 |  |  |  |
| Long Fjord | | 5 | 0.006 | ± 0.004 | | 2 | | 2.800 ± 1.768 | | |  | | 0.002 | ± 0.002 | 2 | 2.400 ± 1.556 |  |  |  |
| Herring Islands | | 21 | 0.010 | ± 0.006 | | 6 | | 5.104 ± 2.578 | | |  | | 0.004 | ± 0.002 | 4 | 4.180 ± 2.163 |  |  |  |
| Swain Group | | 7 | 0.004 | ± 0.003 | | 3 | | 1.714 ± 1.131 | | |  | | 0.003 | ± 0.002 | 4 | 3.904 ± 2.224 |  |  |  |
| Colbeck | | 16 | 0.011 | ± 0.006 | | 14 | | 5.208 ± 2.659 | | |  | | 0.006 | ± 0.003 | 16 | 6.500 ± 3.245 |  |  |  |
| Pointe Géologie | | 18 | 0.010 | ± 0.006 | | 15 | | 4.830 ± 2.472 | | |  | | 0.005 | ± 0.003 | 16 | 5.169 ± 2.625 |  |  |  |
|  | |  |  |  | |  | |  | | |  | |  |  |  |  |  |  |  |
| Weddell seals | | **90** | **0.010** | **± 0.006** | | **38** | | **5.102 ± 2.498** | | | **0.114 ± 1.76x10^-3^** | | **0.005** | **± 0.003** | **40** | **5.658 ± 2.739** | **2.85x10^-2^ ± 4.48x10^-4^** |  |  |
| Subfossil WSs | | 6 | 0.009 | ± 0.006 | | 4 | | 4.466 ± 2.560 | | |  | | 0.005 | ± 0.003 | 4 | 5.133 ± 2.895 |  |  |  |
| Auster | | 21 | 0.024 | ± 0.013 | | 20 | | 15.15 ± 7.040 | | |  | | 0.003 | ± 0.002 | 7 | 2.747 ± 1.504 |  |  |  |
| Amanda Bay | | 24 | 0.027 | ± 0.014 | | 24 | | 17.00 ± 7.824 | | |  | | 0.003 | ± 0.002 | 12 | 2.960 ± 1.594 |  |  |  |
| Pointe Géologie | | 22 | 0.032 | ± 0.016 | | 22 | | 20.28 ± 9.264 | | |  | | 0.003 | ± 0.002 | 10 | 2.960 ± 1.602 |  |  |  |
| Fold Island | | 24 | 0.030 | ± 0.015 | | 24 | | 18.70 ± 8.567 | | |  | | 0.003 | ± 0.002 | 8 | 2.889 ± 1.568 |  |  |  |
|  | |  |  |  | |  | |  | | |  | |  |  |  |  |  |  |  |
| Emperor penguins | | **91** | **0.028** | **± 0.014** | | **90** | | **17.89 ± 8.009** | | | **0.760 ± 4.55x10^-3^** | | **0.003** | **± 0.002** | **26** | **2.857 ± 1.516** | **3.88x10^-2^ ± 3.58x10^-4^** |  |  |
| Subfossil EPs | | 3 | 0.0064 | ± 0.0054 | | 3 | | 4.000 ± 2.725 | | |  | | 0.002 | ± 0.001 | 3 | 2.000 ± 1.511 |  |  |  |
|  |  | | |  |  | |  | |  |  | |  | | | |  |  | |  |

**S3. Bayesian skyline plot for emperor penguins based on cytochrome *b* alone**

**
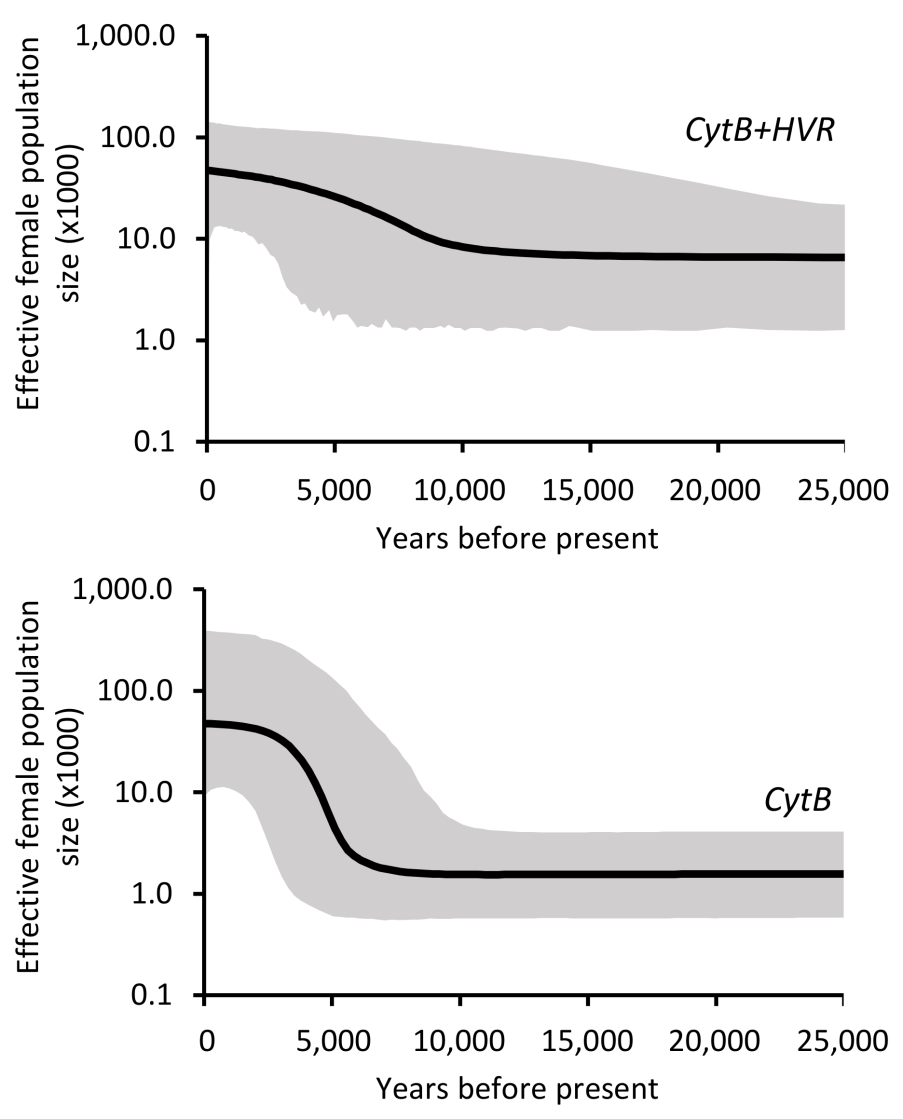
**
